# Using epigenomics data to predict gene expression in lung cancer

**DOI:** 10.1186/1471-2105-16-S5-S10

**Published:** 2015-03-18

**Authors:** Jeffery Li, Travers Ching, Sijia Huang, Lana X Garmire

**Affiliations:** 1 Department of Biomedical Engineering, Johns Hopkins University, Baltimore, MD 21218, USA; 2 Molecular Biosciences and Bioengineering Graduate Program, University of Hawaii at Manoa, Honolulu, HI 96822, USA; 3 Epidemiology Program, University of Hawaii Cancer Center, Honolulu, HI 96813, USA

## Abstract

**Background:**

Epigenetic alterations are known to correlate with changes in gene expression among various diseases including cancers. However, quantitative models that accurately predict the up or down regulation of gene expression are currently lacking.

**Methods:**

A new machine learning-based method of gene expression prediction is developed in the context of lung cancer. This method uses the Illumina Infinium HumanMethylation450K Beadchip CpG methylation array data from paired lung cancer and adjacent normal tissues in The Cancer Genome Atlas (TCGA) and histone modification marker CHIP-Seq data from the ENCODE project, to predict the differential expression of RNA-Seq data in TCGA lung cancers. It considers a comprehensive list of 1424 features spanning the four categories of CpG methylation, histone H3 methylation modification, nucleotide composition, and conservation. Various feature selection and classification methods are compared to select the best model over 10-fold cross-validation in the training data set.

**Results:**

A best model comprising 67 features is chosen by ReliefF based feature selection and random forest classification method, with AUC = 0.864 from the 10-fold cross-validation of the training set and AUC = 0.836 from the testing set. The selected features cover all four data types, with histone H3 methylation modification (32 features) and CpG methylation (15 features) being most abundant. Among the dropping-off tests of individual data-type based features, removal of CpG methylation feature leads to the most reduction in model performance. In the best model, 19 selected features are from the promoter regions (TSS200 and TSS1500), highest among all locations relative to transcripts. Sequential dropping-off of CpG methylation features relative to different regions on the protein coding transcripts shows that promoter regions contribute most significantly to the accurate prediction of gene expression.

**Conclusions:**

By considering a comprehensive list of epigenomic and genomic features, we have constructed an accurate model to predict transcriptomic differential expression, exemplified in lung cancer.

## Background

Epigenetics is a rapidly expanding biological field recently. Aberrant epigenetic modifications are associated with many different diseases including cancers and neurodevelopmental disorders [1]. Much work has demonstrated that epigenetic regulation plays an important role in gene expression, among other mechanisms such as transcription factor regulation. Advances in high throughput methods such as methylation arrays, CHIP-Sequencing, gene expression microarray and RNA-Sequencing have enabled researchers to better understand the relationship between epigenetic modification and gene expression at the genome scale. Coupling with the progress in experimental methodology, we have witnessed a wealthy growth of bioinformatics tools to analyze the epigenetics patterns [2-4].

DNA methylation and histone modification are two major mechanisms of epigenetic regulation. The most widely researched type of DNA methylation in human is the cytosine methylation of CpG islands and their associated regions, such as CpG shores [5]. CpG methylation occurs genome-wide in regions related to protein coding genes (promoters, exons, UTRs, etc.) as well as in certain intergenic regions. It has been shown that CpG methylation tends to occur in promoters located upstream of the transcription starting site [6], and increased methylation (hypermethylation) in the promoter is negatively associated with the gene expression level[1]. On the other hand, CpG methylation in gene bodies seems to be positively associated with gene expression [1]. In cancers cells, massive global loss of DNA methylation (hypomethylation) has been observed, and such hypomethylation in promoters can activate aberrant expression of oncogenes [7]. Much new information has been gained through the recently developed methods, such as Illumina Infinium HumanMethylation450 arrays that enable the detection of CpG methylation throughout the different locations associated with over 99% of protein coding genes.

Histone modification is another type of important epigenetic modification [1]. Histones are the core of nucleosomes that DNA sequences wrap around. All histones are subject to some level of methylation or acetylation, which would either open up or close the local chromatin structures to enable or repress gene expression. Among them, Histones 3 (H3) has various kinds of methylation and they serve as well-studied markers for gene expression status. For example, Histone 3 Lysine 4 tri-methylation (H3K4Me3) in the promoter region is an indicator of active gene transcription, and Histone 3 Lysine 36 tri-methylation (H3k36me3) is associated with transcription elongation. Oppositely, Histone 3 Lysine 27 tri-methylation (H3k27me3) may repress gene expression. Even more complicated, histone modification markers interact with DNA methylation, and the consequent patterns of gene expression are the combined effects of their interactions. The genomic assays such as CHIP-sequencing have enabled the generation of large amount of histone modification data.

Although integrative analyses on gene expression and epigenetics regulation abound throughout the literature [8,9], it is our observation that quantitative models which use epigenetic information to accurately predict the up or down regulation of gene expression are currently lacking. A frequent question that a biologist would ask, when methylation data are available but the gene expression data are missing, is how the epigenetic changes of a gene may affect the expression of this gene to be either up or down regulated. This report is aimed to fill in this gap, and provide the users with a model that allows them to estimate the consequence of epigenetic modification on gene expression, when the data for the latter are not available. Towards this goal, we have built a classification predictor for gene expression using the machine learning approach. This model examines a large set of CpG methylation data, histone modification data and genome data, and accurately predicts differential expression of RNA-Seq transcriptome by taking advantage of the publicly available data from the TCGA Project (lung cancer) and the ENCODE project.

## Methods

### Data sets

Several types of high throughput data were used to extract features or classification responses. These include the CpG methylation array data from 50 paired cancer and adjacent normal tissues, three types of histone marker CHIP-Seq data from cancer and normal cell lines, genomic nucleotide sequence and conservation data, and RNA-Seq expression data from samples that have coupled methylation data.

### Data processing

#### Methylation data

The Cancer Genome Atlas (TCGA) Methylation data from Illumina's Infinium HumanMethylation450 Beadchip (Illumina 450k) were used to extract CpG methylation related features, according to their annotation file. The genomic coordinates of CpG, their exons and coding regions were obtained from the Illumina annotation file. Since the annotation file only provided information of transcripts, exons, and coding DNA sequences (CDS), we re-annotated the protein coding genes using the Illumina iGenomes hg19 Refseq annotation in order to extract more comprehensive information from other regions of the transcripts: all introns (with special categories for the first and last intron), as well as first and last exons, untranslated regions in the 5' and 3' direction (5' UTR and 3' UTR, respectively), and a "single exon" or "single intron" designation for transcripts that only had a single exon or single intron.

#### Histone data

Three sets of histone marker CHIP-Seq data, H3k4me3, H3k27me3, and H3k36me3, were considered from two cell lines: A549 cell line (0.2% EtOH treatment) from the lung carcinoma tissue, and SAEC normal lung epithelial cell line (no treatment). Raw CHIP-Seq data were downloaded from the Broad Institute/Bernstein Lab at the Massachusetts General Hospital/Harvard Medical School and the University of Washington in collaboration with the ENCODE project via the UCSC genome browser at http://genome.ucsc.edu. [10,11]. The raw reads were processed in-house to ensure consistency of all normalization procedures. Raw data were first aligned to hg19 using bowtie2 [12], followed by removal of duplicated reads using the Samtools toolkit (specifically, the "rmdup" tool) [13]. The aligned reads were intersected with the relevant segments of the transcript as annotated in the previous section, using the Bedtools toolkit (specifically, the "multicov" tool) [14]. A custom R script was used to normalize the data over total number of reads after removing PCR duplicates.

#### Human genome data

Nucleotide composition data were extracted from hg19 genome FASTA files downloaded from the UCSC genome browser. Conservation scores across three classes of species: vertebrates, primates, and placental animals, were also considered. PhastCons46Way scores were downloaded from the UCSC genome browser [11,15]. Conservation scores were then intersected with the relevant segments of the transcripts using a custom Perl script, in order to extract conservation features.

#### RNA-seq data

RNA-Seq gene expression data from lung cancer samples with coupled CpG methylation data were downloaded from TCGA Research Network: http://cancergenome.nih.gov. Lung adenocarcinoma and lung squamous cell carcinoma data were combined for this project, as they are two subtypes of non-small cell lung cancer. Differential expression analysis was done with the *DESeq2 *package in R [16]. In cases where multiple transcripts are mapped to the same Refseq ID, the geometric mean of the differential expression results was used to represent the gene level expression. In the case that any of these read counts was zero, the counts from all transcripts were artificially increased by one in order to calculate the geometric mean, followed by final subtraction of one. The expression of a gene was then classified as binary outcomes: either up-regulated or down-regulated, once it passed two thresholds: 1) having an adjusted p value < .05 after Holm's multiple hypothesis test [17] and 2) having an absolute value of log2 fold change greater than 1. As a result, 2874 genes were selected as "differentially expressed" genes.

### Feature extraction

The extracted features are categorized into four major sub-groups. All features were considered on a segment-wise basis (see *Data Processing*), unless noted otherwise.

#### CpG Methylation features

Differential expression of the methylated CpG sites was processed using the *limma *library in R. Specifically, the function *toptable *was used to determine the log fold change (*logFC*) between the cancer and normal tissues as well as the average methylation (*avgMval*) of each CpG site across the two types of tissue [18]. A positive logFC indicates hypermethylation whereas a negative logFC indicates hypomethylation. Additional segment-based features were also considered. These include the number of hypermethylated (*numHyper*) and hypomethylated probes (*numHypo*) on a segment of a given transcript. For example, *first_exon_numHyper *refers to the number of hypermethylated probes on the first exon. Two other types of features are the average of *logFC *and *avgMval *of all CpG probes on a segment of the transcript, e.g. the average *logFC *of all probes on the first exon of a given transcript (*first_exon_avglogFC*).

Special effort was paid to compute distances of CpG probes to exon-exon junctions. Given that one or more CpG sites may exist on the individual exon segments of a transcript (including the first and last exons), transcript-level maximum, minimum and average distances of any hyper/hypo-methylated probe to the nearest 5' or 3' exon-exon junction were computed (*maxHypoTo5, minHypoTo5, avgHypoTo5, maxHypoTo3, minHypoTo3, avgHypoTo3, maxHyperTo5, minHyperTo5, avgHyperTo5, maxHyperTo3, minHyperTo3, and avgHyperTo3*).

#### Histone marker modification features

After the alignment of raw histone marker data (see Data Processing), the aligned histone marker reads were intersected with the segments of each transcript using the *multicov *function from the BEDTools package [19]. The histone reads were then normalized per 1000 bp length of each segment per 1 million aligned read library. Similar to the CpG methylation features, the histone marker modification features were extracted on a segment-by-segment basis. Initials are used to represent the individual cell lines where the features come from: *A *for the A549 cell line and *S *for the SAEC cell line. Following the initial is a number representing the specific histone H3 methylation marker: *4 *for H3k4me3, *27 *for H3k27me3, and *36 *for H3k36me3. As a result, features are named as segment_cell type and histone modification type (e.g. *first_exon_A4*). In order to compare histone modification between the cancer and non-cancer cell types, the differences of the reads between them were divided by the average of the two (e.g. a feature named *first_exon_A4_minus_S4_divavg*).

#### Nucleotide features

In each segment of the transcript, four different types of nucleotide features were extracted: single nucleotide composition, dinucleotide composition, trinucleotide composition, and the length of each segment. Nucleotide sequences of Hg19 reference genome were processed using the Biostrings library in R [20].

#### Conservation features

Conservation score per segment was calculated as the arithmetic mean of the conservation score per nucleotide in that segment. Three separate sets of conservation scores with different comparative species were extracted from UCSC genome browser - vertebrate, primate, or placental. Thus, features such as *first_exon_vertebrate *emerge from this set.

### Feature selection

Three feature selection methods were considered: Correlation Feature Selection (CFS) [21], Gain Ratio [22] and ReliefF [23].

CFS is based on mutual information, a non-linear measure of correlation. CFS selects an approximately optimal set of features to maximize the relevance and minimize redundancy. Relevance is the correlation of a feature to the class (up-regulated or down-regulated gene expression) measured by mutual information, whereas redundancy is the correlation between two features. Redundancy between selected features is minimized to keep the number of selected feature small.

The Gain Ratio is an improved method of Information Gain (IG). Both feature selection methods employ a decision tree in their respective algorithms. The Gain Ratio, by name, is a ratio of IG, but it overcomes the bias of IG which favors features with more data.

ReliefF is an improved feature selection method from Relief. Relief uses the Manhattan distance of its nearest hit and miss from a random instance to continuously update a weight vector, which is then used to calculate a relevance score. Features above a certain relevance threshold are considered "selected" [24]. ReliefF improves on Relief in several ways, including two improvements particularly important for this report. First, ReliefF extends Relief to be able to handle incomplete or partial data. Second, ReliefF searches for *k *near-hits and near-misses and takes their averages, as opposed to one nearest hit or miss from Relief. *k *=10 was sufficient to obtain satisfactory results [23].

CFS is the only method that has a built-in system for selecting the number of features. Gain Ratio and ReliefF both work as ranker systems, meaning every input has a matching respective ranked output. In order to ensure fairness between feature selection methods, we matched the numbers of selected features from Gain Ratio and ReliefF to be the same as determined by CFS.

### Model evaluation

The data were split into training and testing sets. The training set constituted 80% of the up-regulated and down-regulated genes, and the testing set constituted the remaining 20% genes. The training data set underwent 10-fold cross validation on various combinations of feature selection and classification methods, in order to obtain the best model.

After determining the best model, two sets of drop-off tests were conducted. The first set of tests considered the effect of data types, including nucleotide composition, histone markers and methylation data, on the performances of sub-models. The second set of drop-off tests considered the effects of different segments on transcripts, including gene body, exons, introns, UTRs, TSS1500 and TSS200, on the methylation CpG methylation data based sub-models. For each drop-off test, a set of features was removed from the original input features prior to the feature selection and classification. Subsequently the same ReliefF feature selection and RF classification for the drop-off tests were performed as described in the previous *Feature Selection *section.

### Software

Weka 3 data mining software [25] was used for feature selection, classifier training and evaluation. Various R packages were used, including Corrplot for generation of the correlation matrix [26], and ROCR for ROC curves [27]. The classification model is available at: https://github.com/lanagarmire/epiPredictor

## Results

### Summary of input data and features

**Figure 1:**
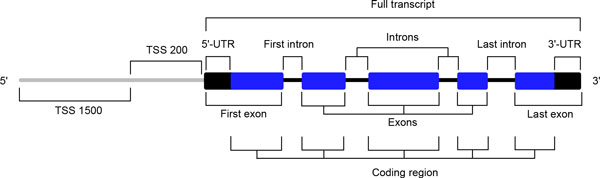
**Segments associated with protein coding genes**. Features considered to predict differential gene expression are depicted on a segment-by-segment basis. Segments are determined based on the annotations of Illumina Infinium Human Methylation 450K Beadchip Array, with augmentations on segments located in gene bodies. From 5' to 3' end of the protein coding genes, listed are transcription starting sites (TSS) upstream up to 1500 bp (TSS 1500) and 200 bp (TSS 200), first exon which may include 5' UTR, first intron, exon body, last intron, and last exon which may include 3' UTR. A full transcript region is determined as the UTRs and coding region together.

Four types of input data were used to extract the features including the Illumina 450K CpG methylation array data from cancer and normal tissues, three types of histone H3 marker CHIP-Seq data from cancer and normal cell lines, genomic nucleotide sequence and conservation data, and RNA-Seq gene expression data from samples with coupled CpG methylation data. In total, we calculated 1424 features and summarized the features by column. These features can be divided into two categories (Table 1): (1) data type based features, including average CpG methylation, average methylation log fold change, number of hyper/hypo-methylated probes, mono-nucleotide, di-nucleotide and tri-nucleotide composition, histone H3 methylation CHIP-Seq reads, and Phastcon conservation scores; (2) segment based CpG methylation features from Illumina 450K BeadChip annotations: upstream of the transcription start site (TSS) 1500, TSS200, 5' and 3' UTRs, exon/intron body, first and last exon/intron, single exon/intron and full transcript (Figure 1 and Table 1).

**Table 1:** The list of all features considered prior to feature selection

	Average M value (Methylation)	Average Log Fold Change (Methylation)	Number of hypermethylated probes	Number of hypomethylated probes	Single nucleotide composition	Dinucleotide composition	Trinucleotide composition	Length of segment	Histone reads	Histone read comparisons (difference of reads/average of reads)	Conservation scores (PHASTCONS)	
TSS 1500	TSS1500_avgMval	TSS1500_avglogFC	TSS1500_numHyper	TSS1500_numHypo	TSS1500_A	TSS1500_AA	TSS1500_AAA	--	TSS1500_S27	TSS1500_A27_minus_S27_divavg	TSS1500_vertebrate	

TSS 200	TSS200_avgMval	TSS200_avglogFC	TSS200_numHyper	TSS200_numHypo	TSS200_A	TSS200_AA	TSS200_AAA	--	TSS200_S27	TSS200_A27_minus_S27_divavg	TSS200_vertebrate	

UTR5	UTR5_avgMval	UTR5_avglogFC	UTR5_numHyper	UTR5_numHypo	UTR5_A	UTR5_AA	UTR5_AAA	UTR5_length	UTR5_S27	UTR5_A27_minus_S27_divavg	UTR5_vertebrate	

First exon	first_exon_avgMval	first_exon_avglogFC	first_exon_numHyper	first_exon_numHypo	first_exon_A	first_exon_AA	first_exon_AAA	first_exon_length	first_exon_S27	first_exon_A27_minus_S27_divavg	first_exon_vertebrate	

First Intron	first_intron_avgMval	first_intron_avglogFC	first_intron_numHyper	first_intron_numHypo	first_intron_A	first_intron_AA	first_intron_AAA	first_intron_length	first_intron_S27	first_intron_A27_minus_S27_divavg	first_intron_vertebrate	

Exon Body	exon_avgMval	exon_avglogFC	exon_numHyper	exon_numHypo	exon_A	exon_AA	exon_AAA	exon_length	exon_S27	exon_A27_minus_S27_divavg	exon_vertebrate	

Intron Body	intron_avgMval	intron_avglogFC	intron_numHyper	intron_numHypo	intron_A	intron_AA	intron_AAA	intron_length	intron_S27	intron_A27_minus_S27_divavg	intron_vertebrate	

Coding Region (CDS)	CDS_avgMval	CDS_avglogFC	CDS_numHyper	CDS_numHypo	CDS_A	CDS_AA	CDS_AAA	CDS_length	CDS_S27	CDS_A27_minus_S27_divavg	CDS_vertebrate	

Last Intron	last_intron_avgMval	last_intron_avglogFC	last_intron_numHyper	last_intron_numHypo	last_intron_A	last_intron_AA	last_intron_AAA	last_intron_length	last_intron_S27	last_intron_A27_minus_S27_divavg	last_intron_vertebrate	

Last Exon	last_exon_avgMval	last_exon_avglogFC	last_exon_numHyper	last_exon_numHypo	last_exon_A	last_exon_AA	last_exon_AAA	last_exon_length	last_exon_S27	last_exon_A27_minus_S27_divavg	last_exon_vertebrate	

UTR3	UTR3_avgMval	UTR3_avglogFC	UTR3_numHyper	UTR3_numHypo	UTR3_A	UTR3_AA	UTR3_AAA	UTR3_length	UTR3_S27	UTR3_A27_minus_S27_divavg	UTR3_vertebrate	

Full Transcript	fullTranscript_avgMval	fullTranscript_avglogFC	fullTranscript_numHyper	fullTranscript_numHypo	fullTranscript_A	fullTranscript_AA	fullTranscript_AAA	fullTranscript_length	fullTranscript_S27	fullTranscript_A27_minus_S27_divavg	fullTranscript_vertebrate	

Single Exon	single_exon_avgMval	single_exon_avglogFC	single_exon_numHyper	single_exon_numHypo	single_exon_A	single_exon_AA	single_exon_AAA	single_exon_length	single_exon_S27	single_exon_A27_minus_S27_divavg	single_exon_vertebrate	

Single Intron	single_intron_avgMval	single_intron_avglogFC	single_intron_numHyper	single_intron_numHypo	single_intron_A	single_intron_AA	single_intron_AAA	single_intron_length	single_intron_S27	single_intron_A27_minus_S27_divavg	single_intron_vertebrate	

Total Features	14	14	14	14	56	224	896	12	84	42	42	1412



Exon-exon junction distances:	Maximum distance to 5' end	Maximum distance to 3' end	Minimum distance to 5' end	Minimum distance to 3' end	Average distance to 5' end	Average Distance to 3' end						

Hypermethylated	maxHyperTo5	maxHyperTo3	minHyperTo5	minHyperTo3	avgHyperTo5	avgHyperTo3						

Hypomethylated	maxHypoTo5	maxHypoTo3	minHypoTo5	minHypoTo3	avgHypoTo5	avgHypoTo3						

Total Features	2	2	2	2	2	2	12					

### Model selection and evaluation

**Figure 2:**
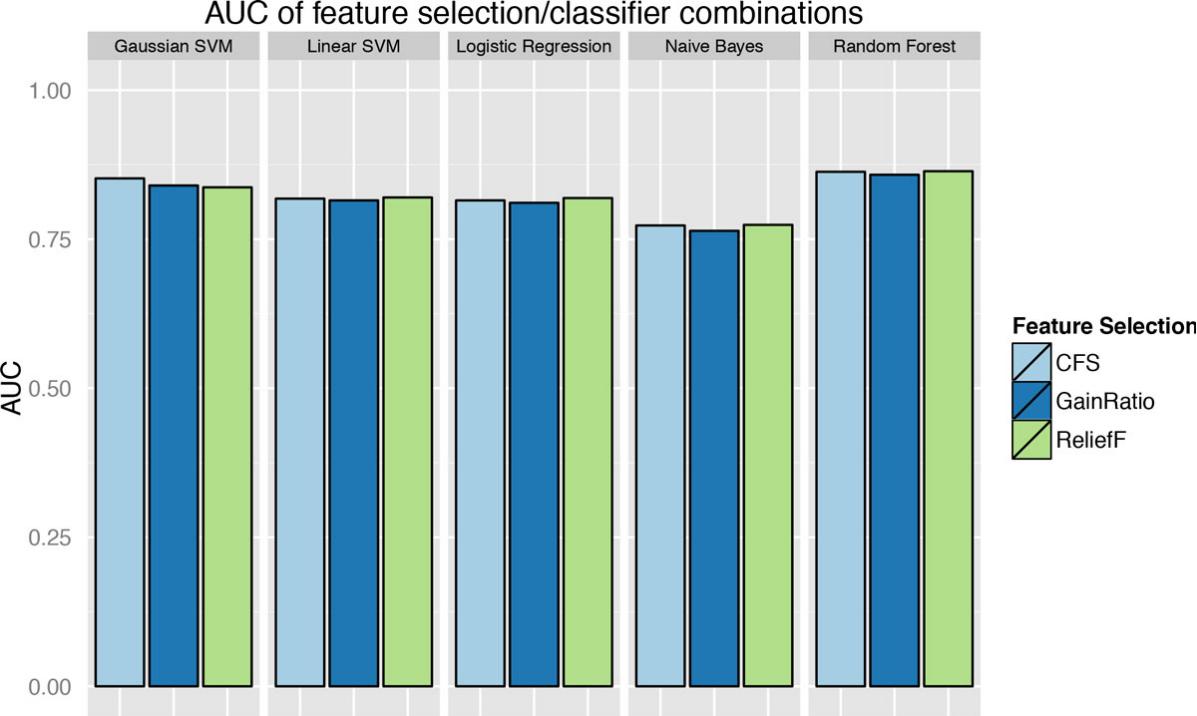
**Performance comparison of models with various feature selection and classification methods**. The Areas Under the Curve (AUC) of ROC are used as the metric to compare the performance of models with different combinations of feature selection (CFS, Gain Ratios and ReliefF) and classification (Gaussian SVM, Linear SVM, Logistic regression, Naïve Bayes and Random Forest), on the training data with 10 fold cross-validation. The model with ReliefF based feature selection and Random Forest classification is selected as the best model.

The model uses 2298 gene data points in the training set, with an additional 576 genes kept in the testing set. Three different feature selection methods were evaluated in combination with five classification methods, using 10-fold cross-validation on the training data set (Figure 2). The three feature selection methods are: correlation-based feature selection (CFS), ReliefF, and Gain Ratio. In most cases with combined classification methods, except for Gaussian SVM, ReliefF gives the best AUCs among the three feature selection methods. Among the five classification methods that we considered, namely Gaussian SVM, linear SVM, Logistic Regression, Naïve Bayes and Random Forest, the two non-linear methods (Gaussian SVM and Random Forest) show superior performances to the other linear classifiers (Logistic Regression, linear SVM, and Naïve Bayes). This indicates that interactions exist among the selected features. However, the differences are not very big, suggesting that the decision boundary is close to linear. Given that the model based on ReliefF feature selection and Random Forest classification gives the best AUC of 0.864, it is selected as the best model for the rest of the project. Similarly, a ReliefF and Random Forest based model has the best predictive performance on the 20% holdout data set, with an AUC of 0.836.

### Analysis of selected features

**Figure 3:**
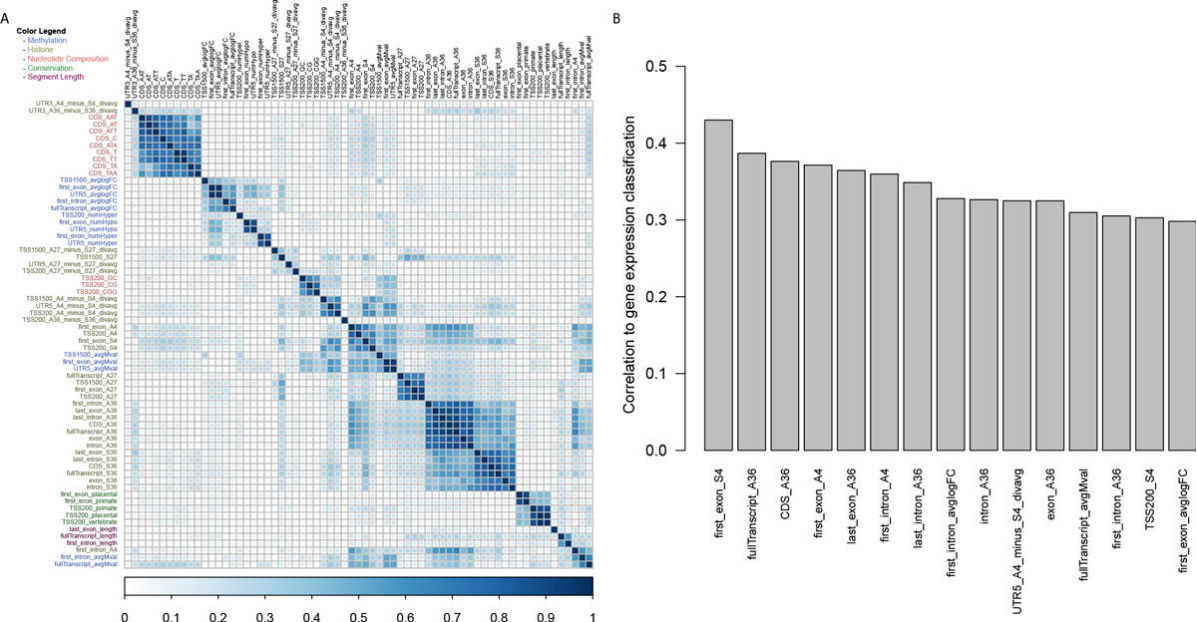
**Top fifteen features from the best model**. (a) The clustering results on the absolute values of Pearson's correlation coefficients from 67 selected features by the best model. The names of different type of features are labeled by different colors. Note: the length of a segment is listed out separately. (b) List of top fifteen features selected by ReliefF feature selection and sorted by their correlation to the classification of differential gene expression.

A total of 67 features are selected by the best model, spanning all four types of genomic and epigenomic data. We first explored the relationship among the selected features. Using hierarchical clustering on absolute correlation values between features (Figure 3A), we found that the selected features tend to cluster by the data type, as expected. For example, the conservation features in the coding regions (CDS) are grouped together, and so are most methylation features. As expected, the CpG islands within the promoter are very important for the prediction of gene expression, as demonstrated by the three selected and highly correlated features CG composition features, *TSS200_GC, TSS200_CG and TSS200_CGG*.

The largest group of selected features is the histone modification features (32 features), followed by the methylation features (15 features) (Additional File 1). The selected features underscore the importance of histone epigenetic modification in the regulation of gene expression. Likewise, the importance of methylation features is evident, especially for the featured arising from TSS, 5' UTR and first exons. Interestingly, several methylation features (*TSS1500_avgMval, first_exon_avgMval and UTR5_avgMval*) are clustered with histone modification features, suggesting collinearity between these two types of features, as shown by others [28,29].

On the other hand, when features are categorized by location relative to the transcripts (Additional File 1), the TSS200 has the most number of features (13 features), and TSS1500 has 6 features selected for this region. Together, the promoter comprises 28% of all the selected features. This confirms the previously well-known importance of the promoter region for the epigenetic regulation of gene expression [30,31]. Additionally, CDS has the second highest number of features being selected, highlighting its significance in regulating gene expression [30].

We also calculated the correlation of each feature to gene expression and plotted the top 15 features most relevant to gene expression prediction (Figure 3B). None of the features have correlations higher than 0.45, suggesting that no single feature is a dominant predictor for gene expression. These features are either histone modification (11 features) or methylation features (4 features), consistent with the previous observation on the significance of these two types of features.

### Evaluation of features by data type

**Figure 4:**
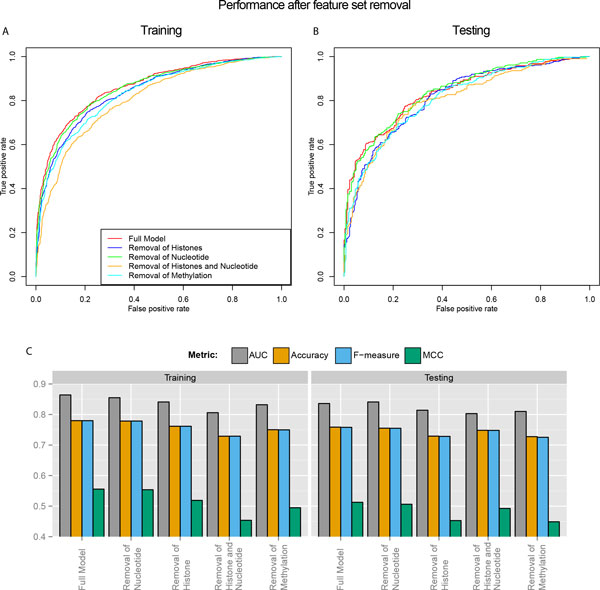
**Evaluation of features generated from various data types**. (a-b) Effects of feature set drop-off on ROC curves from the 10-fold cross-validation training set (a) and testing set (b). (c) Effects of feature set drop-off on other four metrics: AUC, Accuracy, F-measure and MCC, in the training set and testing set.

To determine the contribution of different types of features to gene expression, we tested the performance of models when a subset of features from the same data type were dropped. We present the results of four measures of model performance: AUC, accuracy, F-measure and Matthew's correlation coefficient (MCC) (Figure 4). Dropping any individual feature set of nucleotide composition, histone modification or CpG methylation, did not seem to have a large effect on the model performance, indicating that there is redundancy between feature sets. The sub-model performance for the dropping-off of a single feature set from the full model is in the following order: nucleotide composition removal > histone modification removal > CpG methylation removal. Thus dropping methylation features had the largest effect among individual feature set, as the AUC decreases from 0.864 in the full model to 0.832 in the training set, as well as from 0.836 to 0.810 in the testing set. Likewise, MCC, upon single feature set drop-off, shows the largest proportional change among the four performance measures, and decreases from 0.56 to 0.49 on the training set and 0.51 to 0.45 on the testing set.

We also compared the effect of removing both nucleotide and histone features on model performance, as compared to removing either of them alone. As expected, removing both nucleotide and histone features gives the lowest AUCs, lowest accuracies and lowest F-measures in both training and testing sets. However, it leads to higher MCC than removing just histones does in the testing set. This suggests that there might be some overfitting with regards to the nucleotide feature set, which accounts for the majority (83%) of features prior to feature selection.

### Evaluation of CpG methylation features by locations relative to transcripts

**Figure 5:**
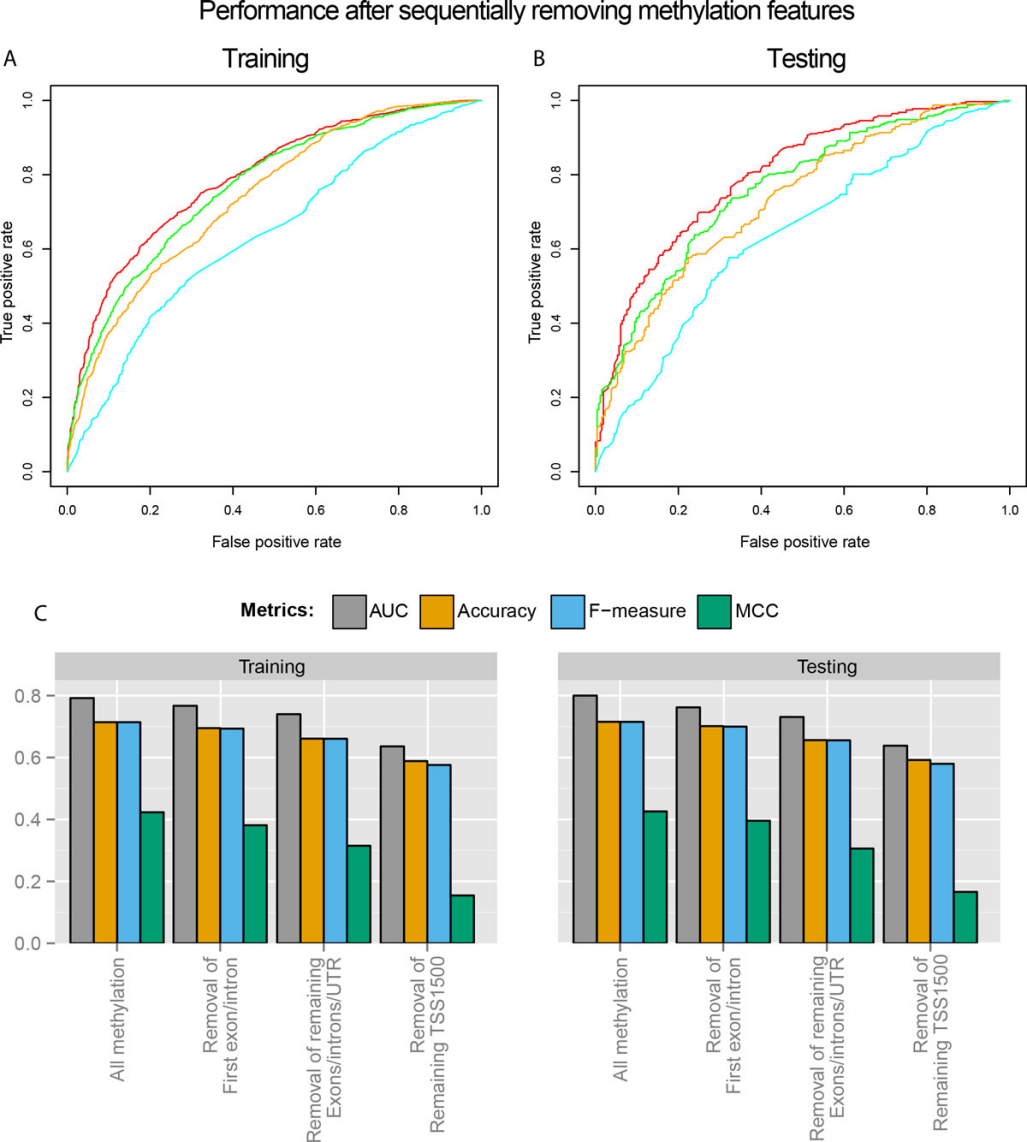
**Evaluation of methylation features by segment**. (a-b) Effects of segment-based methylation feature set sequential drop-off on ROC curves from the 10-fold cross-validation training set (a) and testing set (b). (c) Effects of segment-based methylation feature set sequential drop-off on other four metrics: AUC, Accuracy, F-measure and MCC, in the training set and testing set.

Given that removing methylation features causes the most reduction of model performance among the single feature set drop-off (Figure 5), we next asked the question of the relative importance of each methylation feature categorized by genomic location. We performed drop-off tests by sequentially removing features in each genomic location category. We first removed the features from the first exons and first introns (as they are close to the TSS), then from gene bodies including exons, introns, and UTRs, and lastly from TSS1500 region such that only TSS200 features were kept. At each step, we re-performed feature selection and model construction, using the remaining methylation features.

All categories of genomic locations provide relevant useful information that contributes to better prediction of gene expression, as each of the sequential feature set drop-off decreased the performance of the model in both training and testing sets. Compared to the removal of first exon and intron regions, and removal of the UTRs and the rest of the gene body, removal of TSS1500 leads to the largest reduction in all four metrics, confirming the importance of the promoter region in regulating gene expression. Even when only TSS200 features are considered, an AUC of 0.638 and 0.636 are obtained in the testing and training sets respectively, suggesting that CpG methylation status in TSS200 is still somewhat predictive of gene expression. However, a more accurate prediction using methylation features should arise from all locations associated with the transcripts.

## Discussion

### The need to build predictive models of gene expression from epigenomics data

Although currently integrative analyses between gene expression and epigenetic modification exist, we have found that quantitative models using epigenetic information to accurately predict the up or down regulation of gene expression are currently lacking. There are often cases where researchers can only obtain reliable epigenetics data, but not gene expression data. For example, if the samples are archived and processed by FFPE (Formalin-fixed, paraffin-embedded), one can still perform epigenomics measurements, but not the gene expression experiments due to the degradation of mRNA in the samples. More importantly, a predictive method such as ours can efficiently facilitate the bench scientists to narrow down the candidate lists and conduct gene expression validation, especially when the epigenetics information is the only data handy.

### Selected features and their relevance to gene expression

All four types of data (CpG methylation, histone H3 modification, nucleotide sequence and conservation) exist in the 67 features that are selected by the best model, indicating that all of them contribute to the accurate prediction of gene expression. Moreover, selected features of the same data type tend to cluster together on the correlation matrix among the features, suggesting that the relationship within the same data type is closer than the relationship between different data types. As expected, histone modification and CpG methylation features are the largest two groups among the four types of data, signifying their importance to predict gene expression. Since nonlinear classification methods perform slightly better than linear classification methods, it suggests that interactions do exist between different types of data. This is supported by numerous literatures that enzymes responsible for CpG methylation also interact with histone modification events [32,33].

Besides the value of predicting gene expression, our models also provide insights into the relative importance of different epigenomics/genome data, as well as the genomic locations. We found that CpG methylation features have more predictive values for differential gene expression, compared to the three types of histone H3 modification data. Although other kinds of histone modification data can also be obtained to increase the predictive values of histone modification data, it is much more costly to obtain them relative to the CpG methylation data (the cost of CHIP-Seq on each of the histone modification marker is similar to an entire CpG methylation array). Therefore, practically speaking, when the budget is a constraining factor, we suggest that assays on CpG methylation should be considered with priority in predicting differential gene expression. Moreover, the results of our models demonstrate that all genomic locations relative to each transcript, including promoters, exons and gene bodies, provide useful information to predict gene expression alternation. Although the CpG methylation signals from the promoters region are more important, the methylation signals from other regions, such as exons, introns and UTRs are indicative of changes in the gene expression as well.

Worth noticing, a lot of features that are extracted on methylation and histone modification are naturally based on the annotations from Illumina 450K array platform for DNA methylation. There may be bias on the number of features that are hand coded in the model. To address potential issue, we changed TSS200_GC to TSS150_GC in our model and obtained an AUC = .861 (compared to 0.864) for cross fold validation on the training set and an AUC = .834 (compared to 0.836) for the testing set. Therefore, we think the bias due to relying on the nomenclatures from Illumina's annotation is small.

### Limitations and future directions

We should point out that our current model does not include all histone modification data, but only three widely used methylation markers on histone H3 (H3K4Me3, H3K27Me3 and H3K36Me3). Moreover, the histone H3 data are drawn from ENCODE cell lines, since the TCGA samples do not have such data. The heterogeneity of the sample resources could affect the accuracy of the model. When more histone marker data coupled with DNA methylation and RNA-Seq data become publicly available for lung cancer, we can include them to achieve a better model. In the ideal setting, we would like to build a predictive model that has multiple types of epigenomics data obtained from the same samples. Another potential concern is overfitting in the classification model. However, we split the dataset into training and independent testing subsets and show the model performs comparably well on the holdout testing subset. We believe that the model can be replicated if we can identify paired RNA-Seq and methylation data. In fact, we had originally built this model on a private data set, which also achieved an AUC of more than 0.80. Additionally, Figure 4 and 5 both indicate that our approach does not suffer a significant over-fitting problem using the TCGA data, and show the dominant efforts of histone modification and CpG methylation, which yield an up/down gene expression prediction with an AUC>0.80. Currently the model uses lung cancer data, and it will be interesting find out more general epigenetic predictors for differential gene expression in other cancers as well. Lastly, we should point out that regulation of gene expression is complex, including other mechanisms mediated by transcription factors, microRNA, non-coding RNAs etc. The fact that AUCs hover between 0.80-0.90 ranges could be well due to the fact that features from these other mechanisms are not considered in the current epigenetics model. To increase the accuracy, a more complex model that takes into account of all these events should be constructed.

## Conclusions

A new model based on epigenomics data is proposed to predict transcriptome-level differential gene expression in lung cancers. Dropping-off feature sets by data type shows that CpG methylation features are most important for the prediction. Furthermore, methylation features on all genomic regions relative to protein coding genes contribute to the differential gene expression, within which promoter regions are most important.

## Competing interests

The authors declare that they have no competing interests.

## Authors' contributions

LXG envisioned the project and supervised the work. TC initiated the project. JL and TC designed and implemented the project. SJ assisted the project. JL, TC and LXG wrote the manuscript. All authors have read, revised and approved the final manuscript.

## Supplementary Material

Additional file 1 **Table S1**. Selected 67 features in the best model sorted by category and their frequency Click here for file

## Declarations

Publication charges for this article were funded by NIH/NIGMS P20 COBRE GM103457, NIH/NIEHS K01 ES025434-01 and Hawaii Community Foundation.

This article has been published as part of *BMC Bioinformatics *Volume 16 Supplement 5, 2015: Selected articles from the 10th International Symposium on Bioinformatics Research and Applications (ISBRA-14): Bioinformatics. The full contents of the supplement are available online at http://www.biomedcentral.com/bmcbioinformatics/supplements/16/S5.
